# Tenants’ residential mobility in Switzerland: the role of housing functions

**DOI:** 10.1007/s10901-021-09874-5

**Published:** 2021-06-24

**Authors:** Anna Pagani, Ivo Baur, Claudia R. Binder

**Affiliations:** Laboratory for Human-Environment Relations in Urban Systems (HERUS), Environmental Engineering Institute (IIE), School of Architecture, Civil and Environmental Engineering (ENAC), École Polytechnique Fédérale de Lausanne (EPFL), Lausanne, Switzerland

**Keywords:** Housing system, Residential satisfaction, Triggers, Residential preferences, Logit models

## Abstract

The interaction between residential preferences and dwellings is a complex system whose function thus far remains insufficiently explored. In this paper, we investigate housing functions as orchestrators of households’ residential mobility in the context of Swiss rental housing. We propose a theoretical multi-step model and use survey data from 878 Swiss tenants to inspect the model’s linkages. From the statistical analysis, we firstly observe that tenants’ residential satisfaction is more likely to increase when the gap between ideal housing functions and those actually fulfilled by the current dwelling decreases. Secondly, results show that the effectiveness of an event (e.g. a job opportunity) in triggering the move is significantly related to both residential satisfaction and the functions the dwelling fulfils prior to the trigger. Thirdly, findings show that these trigger events can be grouped into three types: radical change, problem-solving and opportunity. With a medium effect size, a radical change was found to bring about the strongest change in housing functions between past and current dwellings. Lastly, in line with the hypothesis that residential preferences vary over the life course, socio-demographic characteristics and tenancy types are found to be significant explanatory variables for households’ ideal housing functions. By disentangling the complexity of the housing system, the proposed multi-step model can be used to integrate households’ preferences with supply-side constraints in agent-based model simulations, thereby contributing to fostering the provision of quality housing, i.e. dwellings able to meet the needs of current and future occupants.

## Introduction

In Switzerland and worldwide, there is an urgent imperative to increase housing quality and adequacy in meeting the needs of current and future inhabitants (Acioly & Horwood, 2011; Lawrence, 2009). In this context, achieving a better understanding of the process by which households match their housing needs to the dwellings available to them is critical. However, the study of the residential mobility process is a complicated endeavour (Clark, 2012; Dieleman, 2001). It involves different geographical scales (i.e. international, national, metropolitan, households; Clark, 2012), or levels (i.e. micro, macro; Mulder & Hooimeijer, 1999; van Ham, 2012), a multitude of disciplinary lenses (Coolen et al., 2002; Lu, 1999; Mulder, 1996; Wong, 2002), and a variety of stakeholders (i.e. the owners, the tenants, the policy-makers; Lawrence, 2009). Moreover, it entails dealing with the delicate interactions of a complex human–environment system that extends beyond the material aspects of dwellings (Lawrence, 2009).

Few scholars have attempted to make sense of this complex system in the Swiss context, where questions relating to habitat remain relatively little addressed (Pattaroni et al., 2009). Among these, Lawrence (2009) introduced the federative concept of *attractiveness*, which lies at the intersection between offer and demand and accounts for both the objective characteristics of housing stock and the multiple perspectives of actors, institutions and households concerning features’ strengths and weaknesses. Greater attractiveness ratings result in higher satisfaction among households and lower residential relocations and vacancy rates (Lawrence, 2009). The recent work of Pagani and Binder (2021) extended this reflection one step further with the introduction of a systems perspective to housing studies. Housing is conceptualized as a system of human and material structures whose behaviour (i.e. residential preferences; dwelling forms) is determined by the system’s *function(s)* (Bossel, 1999; Hester & Adams, 2017; Meadows, 2008). Although their study illustrates a promising application of the notion of housing function to the field of residential mobility, their findings remain at the exploratory level.

With the goal to achieve an enhanced understanding of Swiss households’ residential mobility and thereby contribute to fostering the provision of housing that meets current and future users’ needs, this paper investigates the role of housing functions in the decisions to move and select a new dwelling based on survey data that targets the tenants of a real estate owner and of two of the largest cooperatives in Switzerland. More specifically, we address the following question and sub-questions:

*What role do housing functions play in orchestrating the factors determining the moves of Swiss tenants?*

- Can housing functions be used to understand residential mobility?
- To what extent do housing functions influence which determinants are effective in tenants’ decision to move?
- How are the housing functions of the new dwelling influenced by this decision?
- Do socio-demographic characteristics and tenancy type have an influence on households’ ideal housing functions?

To answer these questions, we proceed as follows. In Sect. 2, we explicate our theoretical framework, first reviewing the key literature on housing functions, residential mobility and previous qualitative research in Switzerland and then operationalizing the findings in a multi-step model that integrates the concept of housing functions in tenants’ relocation process. Sect. 3 introduces the statistical methods used to explore the model, following which the results of the analyses are presented in Sect. 4. In Sect. 5, we discuss the relevance of the results for the wider literature along with their practical applicability, critically review the adopted methods and identify avenues for future research.

## A theoretical framework for tenants’ residential mobility

### Housing functions in the housing system

A system is ‘anything that is composed of system elements’ (Bossel, 1999, p. 20). These elements are connected in a structure, which allows the system to perform specific *functions* in its environment. Systems can be nested within other systems (Meadows, 2008).

According to this definition, the housing system has been conceptualized as being embedded in and structured by a societal system comprising rules and resources (e.g. culture, legislation, financial capital) and an environmental system constituting the natural and technical environment (Binder, 2007; Giddens, 1984; Pagani & Binder, 2021). Encompassed within the environment and society are the human and material subsystems, which are in turn structured by e.g. households’ residential biographies and dwellings’ features, and manifest themselves in different residential preferences and dwelling forms, respectively (Pagani & Binder 2021). These manifestations, also called system’s behaviours, are determined by the *functions* of the housing system. For instance, for given societal and environmental structural elements (e.g. geography, culture), the material behaviour of the function *shelter* can be either a detached suburban house or a basic shelter providing shade from the sun or inclement weather; the function *commodity* can entail a prioritization of convenience (price, location) over quality.

Table 1 illustrates the nine housing functions identified by Pagani and Binder (2021). At the interface between residential preferences and dwelling forms, these functions are introduced by the authors as key elements in the investigation of residential mobility, the process of which is outlined in the following section.

**Table 1:** Housing functions (after Pagani & Binder, 2021)

Function	Definition
Shelter	A refuge, a fortress where one can return to get rest, before going back out ‘into the world’; the ‘homely home’
Security, privacy	A private place mainly for the family's needs. The recreation preferably happens outside
Permanence	A place a person feels they belong or are rooted in
Production, consumption	A place that enables one to perform activities (like eating, laundering, companionship)
Impermanence	A place free from tradition or memory, which reflects one’s life stage
Commodity	A temporary place or a starting point. Maybe attractive for its price or location
Status symbol	A credential for esteem, a place for exhibiting
Self-representation	A place for self-expression, satisfaction of aspirations
Property	A place that belongs to the occupant, of which s/he is entitled to do what s/he wants

### Residential mobility

The housing literature is replete with studies on residential mobility. Despite the variety of conceptualizations of the relocation process, many scholars have shared the assumption that an individual first decides to move and then chooses where to relocate (i.e. the two-stage choice approach; Brown & Moore, 1970; Clark & Onaka, 1983; Mulder, 1996; Mulder & Hooimeijer, 1999; Rossi, 1955). In this section, we concisely illustrate previous research on the determinants of the decisions to move and to select a dwelling, and their mediator: residential satisfaction.

Triggers are the *determinants of the decision to move*. Households ‘do not relocate unless there is some trigger (or even an absolute necessity) causing the benefits of moving to outweigh its costs’ (Mulder & Hooimeijer, 1999, p. 162). Brown and Moore (1970, p. 2) defined triggers as stimuli or stressors provided as continuous sources by the environment and perceived differently among households depending on their ‘tolerance to stress’. In more recent studies, triggers are described as arising not only from the environment but also from the life course trajectories of housing, household, education, and work, whereby a move is caused by or timed in accordance with events related to each (e.g. marriage, divorce, university, promotion; Clark & Lisowski, 2017; Coulter, 2013; Dieleman, 2001; Mulder, 1996; Mulder & Hooimeijer, 1999; Rabe & Taylor, 2010). Thus, a plurality of micro- (e.g. new job location) and macro-level factors (e.g. housing market opportunities) can trigger a move.

The concept of *residential satisfaction* lies between the decision to move and that to select a dwelling. Scholars have largely cited a household’s dissatisfaction with a dwelling in terms of housing attributes, neighbourhood characteristics and accessibility as a motivation for moving, while an increase in residential satisfaction has been demonstrated to be a value attached to relocation (Clark & Onaka, 1983; de Groot et al., 2011; Diaz-Serrano & Stoyanova, 2010; Kearns & Parkes, 2003; Kim et al., 2015; Kwon & Beamish, 2013; Lu, 1998; Marans, 1976; Mulder, 1996; Mulder & Hooimeijer, 1999; Speare, 1974). Starting from the seminal work of Wolpert (1965), Brown and Moore (1970), Galster and Hesser (1981) and Galster (1987), residential satisfaction has been conceptualized and calculated as a function of the gap (also called mismatch, discrepancy, disequilibrium, dissonance) between how much a household needs (i.e. desires, aspirations, preferences) and how much is available (i.e. reality; Clark, 2012; Jansen, 2014; Jiang et al., 2017, 2020; Lu, 1999; Phipps & Carter, 1978). Accordingly, the move can be seen as a process of ‘adjustment’ during which households seek to make the best possible match between where they live and how they ‘want to live’ through the exploration and evaluation of qualities of the built environment (Brown & Moore, 1970; De Jong & Fawcett, 1981; Lu, 1998; Phipps, 1989; Thomas & Pattaroni, 2012).

To assess the ways that households ‘want to live’ corresponds to studying the criteria they make explicit in order to evaluate vacancies—i.e. the *determinants of the decision to select* a dwelling (Marans, 1976). These factors are commonly investigated through the analysis of stated and revealed preferences, the latter of which uses information on actual moving behaviour whereas the former is more widely investigated through desires and moving intentions (Coolen et al., 2002; de Groot et al., 2011; Molin et al., 1996; Mulder, 1996; van Ham, 2012). A number of studies have asserted that residential preferences vary between individuals and over their life course (Booi & Boterman, 2019; Coolen et al., 2002; Lawrence, 2004; Mulder, 1996) and therefore change when a trigger affects it (e.g. following a divorce; Brown & Moore, 1970; Jiang et al., 2017; Kim et al., 2015; Mulder & Hooimeijer, 1999).

From the conceptualization of the decision to move and its determinants, a system of interrelationships emerges that directly and indirectly links triggers to move, residential satisfaction and preferences. This system is embedded in metropolitan (i.e. tenure composition, turnover rate), national (i.e. economic and demographic circumstances), and international scales (i.e. housing policies, wealth, tenure structures; Dieleman, 2001). Therefore, to obtain a greater understanding of the decision system of tenants in Switzerland, we introduce the geographical context of our study in the following section.

### Residential mobility and housing functions in Switzerland

Although Switzerland’s high per capita income makes it among the world’s wealthiest nations, its housing market differs from what might be expected in that it is a country of tenants (Pattaroni et al., 2009; Werczberger, 1997). At the end of 2017, an average of 60% of households lived in rented dwellings, with the highest proportions located in the urban cantons of Basel-Stadt (84%) and Geneva (78%; FSO, 2019). The survival of a viable rental sector is remarkable considering that Swiss rent control legislation has been limiting landlords’ ability to raise rents and evict tenants at will for the last 80 years (with the exception of new constructions or units vacated by their tenants; Werczberger, 1997).

In a country where nearly two-thirds of the population are tenants, the rules governing the tenancy of apartments and buildings permit little-to-no inhabitant participation in shaping their living environment (Rabinovich, 2009). However, housing ‘quality’ and ‘conditions’ are considered very satisfactory, except for a lower than ‘natural’ overall vacancy rate (2.7%), in particular in the cities of Lausanne (0.4%) and Zurich (0.1%; Werczberger, 1997; Wüest Partner, 2020; Zimmermann, 1992). Furthermore, Switzerland offers ways to simultaneously be a tenant and an owner, most notably through the housing cooperative system, in which the oldest cooperatives (also called ‘large’ or ‘open’) act as property developers with a social purpose. These cooperatives are responsible for the financing and management of the housing and its operations in order to ensure affordable rents (Rabinovich, 2009).

The Swiss context offers a promising setting for the study of the relocation process; as in most other European countries, the mobility of Swiss households has been increasing in recent decades (Pattaroni et al., 2009), and tenants, who represent the largest share of Swiss occupants, are more mobile than owners (Clark, 2012; Coulter, 2013; Dieleman, 2001; Kwon & Beamish, 2013; Rossi, 1955).

Pagani and Binder’s (2021) research on housing functions and residential mobility is framed in the above-described context. Based on two exploratory group discussions with tenants in the Swiss cities of Lausanne and Zurich, the authors advanced a set of hypotheses regarding the determinants of the decisions to move and select a dwelling. Concerning the former, they inductively formulated three categories of triggers comprising events emerging from the micro- and macro-context: ‘opportunity’ (e.g. construction of a new building in front of the current one); ‘problem-solving’ (e.g. change in job location); ‘radical change’ (e.g. leaving the parental home). Problems to solve and radical changes are imposed triggers, which were observed to happen and become effective no matter how large the satisfaction of a household with its dwelling was; in contrast, an opportunity was found to be effective only when the household displayed a medium-to-low level of satisfaction. Additionally, the authors observed that these trigger types were more—or less—effective depending on the *function* fulfilled by tenants’ dwellings; for instance, an opportunity was more likely to be identified when the dwelling was perceived as a ‘commodity’ or an ‘impermanent’ place. Concerning criteria for selection of the new dwelling, tenants indicated that the functions fulfilled by the dwelling they were living in at the time of the group discussion (i.e. *current* functions) corresponded to the functions they desired when selecting it (i.e. *ideal* functions). Changes between the functions of the former and current dwelling (i.e. revealed preferences) were reported only following radical changes in tenants’ life course, such as leaving the parental home; conversely, catching an opportunity or solving a problem was not observed to affect the housing function(s) of the dwelling to which tenants moved, but rather to improve the quality of or resolve the issues related to a significant feature (e.g. dwelling size; distance to work).

**Fig. 1:**
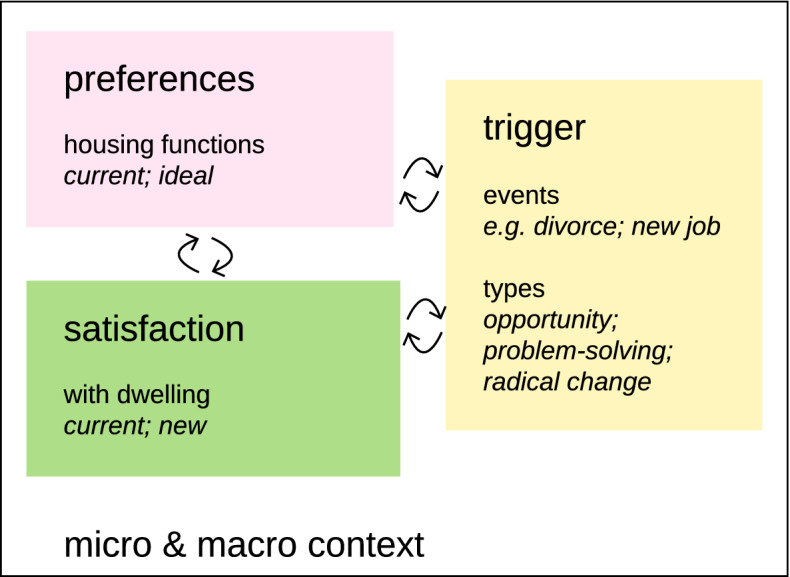
A conceptual framework for the residential mobility of Swiss tenants. Arrows indicate the recursive interactions between triggers to move, households’ residential preferences and their residential satisfaction

To summarize, households’ residential mobility can be described as a process consisting of the decisions to move and where to move. Two types of determinants play a role in the process: triggers events (i.e. determinants to move) and households’ residential preferences (i.e. determinants to select a dwelling). The former can be categorized into three types (opportunity, radical change and problem-solving), whereas the latter can be classified into two types (the ideal and current housing functions). These determinants affect each other, even as they influence and are influenced by the household’s level of satisfaction with its current dwelling and that it considers selecting. The introduced variables are embedded in and shaped by contextual factors at the micro- and macro-levels (e.g. tenants’ life courses, housing market; Fig. 1).

### Hypotheses and model

**Fig. 2:**
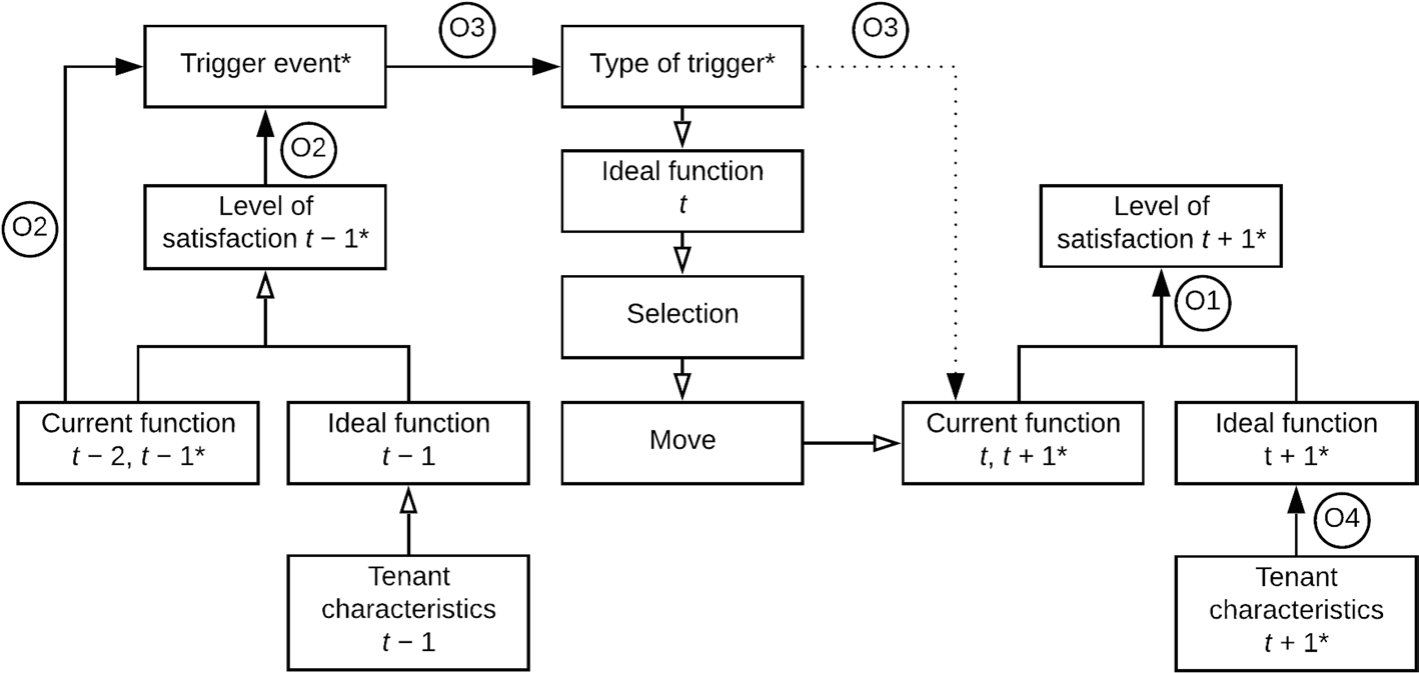
Multi-step model of tenants’ residential mobility. *t* − 2 indicates the time of the past move; *t* − 1 indicates the time prior to the decision to move at time *t*; *t* denotes the time of the decision and relocation; *t* + 1 represents the time following the move. The symbol ‘*’ indicates the measured variables: full arrows show the analysed relationships; dotted lines indicate the proxies used for the analysis; ‘O’ refers to the operational hypotheses tested in this study

Based on the literature reviewed in Sect. 2.3, we propose a set of hypotheses (H) for the residential mobility of tenants in Switzerland. The hypotheses are first operationalized (O) and then summarized in a multi-step model (Fig. 2). Considering the residential tenure under study, the term ‘household’ is used as a synonym of ‘tenant’.

#### H1

Housing functions can be used as proxies for residential attributes (housing, neighbourhood, location) to understand the gap between a household's preferences and reality, i.e. satisfaction with its dwelling. As residential satisfaction plays a key role in both the decision to move and the formulation of households’ preferences, housing functions are relevant for unravelling both processes.

#### ***O1***

*The gap between the housing functions of the ideal and current dwelling is a significant predictor of households’ residential satisfaction.*

#### H2

Housing functions directly and indirectly influence the triggers leading to the move.

#### ***O2***

*The effectiveness of a trigger is significantly related to* (1) *the residential satisfaction prior to the trigger* (*which is itself determined by the gap between current and ideal functions*) *and* (2) *the function*(*s*)* the dwelling fulfils.*

#### H3

Trigger events can be categorized into trigger types, depending on which households readjust their preferences for the new dwelling. More specifically, a change in housing functions occurs only when tenants move in response to the trigger type ‘radical change’.

#### ***O3***

*Trigger events determine trigger types. Changes between the housing functions of past and current dwellings significantly differ across trigger types.*

#### H4

As residential preferences vary over the life course, there is a relationship between households’ characteristics and their ideal housing functions.

#### ***O4***

*Tenants’ characteristics* (*socio-demographics, tenancy types*) *are significant predictors of their ideal housing functions.*

Below, based on the above-advanced hypotheses and their operationalization, we describe the steps of the relocation process explored in this study and illustrated in Fig. 2:

1. At *t* − 1, the size of the gap between a tenant’s ideal housing function and the extent to which such a function is fulfilled by the current dwelling (chosen at *t* − 2) determines the tenant’s residential satisfaction.
2. The level of satisfaction with the dwelling at time *t* − 1 indicates whether a trigger event is effective for the move.^a^
3. The function of the current dwelling at *t* − 1 also indicates the extent to which a trigger event is effective for the move.
4. The trigger events determine which trigger type will lead to the move.
5. Following the trigger and according to its type, the ideal function is updated (time *t*).
6. At time *t,* the tenant selects and moves to a new (current) dwelling, which minimizes the gap between preferences and reality in terms of housing functions.
7. At *t* + 1, the size of the gap between the ideal and current housing functions (chosen at step *t*) determines the tenant’s residential satisfaction.
8. The ideal function at any time step is influenced by the tenant’s characteristics.

^a^Because imposed triggers can occur regardless of households’ satisfaction, we choose the trigger event prior to its categorization into types as the scale of observation.

## Materials and methods

### Survey procedure

To explore the relationships displayed in Fig. 2, we conducted a survey of the tenants of three housing owners: the insurance company and property owner Swiss Mobiliar (Schweizer Mobiliar Asset Management AG), with dwellings all around Switzerland and the housing cooperatives ABZ (Allgemeine Baugenossenschaft Zürich) in the canton of Zurich and SCHL (Société Coopérative d'Habitation Lausanne) in the canton of Vaud. The diversity of housing owners made it possible to consider different types of tenancy and linguistic regions. A draft questionnaire was translated into French and German and reviewed by three laboratories at the Swiss Federal Institute of Technology in Lausanne (EPFL) and Zurich (ETHZ) as well as the housing owners (ABZ, SCHL and Swiss Mobiliar). Following the approval by the Human Research Ethics Committee of EPFL (HREC), the survey was pre-tested by members of the EPFL’s Faculty of the Built Environment (ENAC) and the three housing owners.

The survey was conducted between the 16th of September and the 28th of November 2019. The survey institute LINK collected the data via an online questionnaire addressed to one of the adults in the household who actively influenced the decision to move to her/his current dwelling. Tenants accessed the questionnaire with a personal code shipped by post to 2500 households; those who lacked internet access were given the possibility to participate by phone. The sample was designed based on data provided by the owners and stratified according to four groups: the two cooperatives ABZ and SCHL and the two language regions where Mobiliar dwellings are predominantly located (i.e. French, German). As the initial response rate did not meet our objective, 500 additional tenants were contacted. The final response rate was 32% for a total sample of 968 tenants.

Data were cleaned by inspecting variables (i.e. setting missing values for outliers) and cases (i.e. suppressing cases when answers had a standard deviation of 0 across a block, e.g., tenants who always replied ‘neither, nor’), thereby resulting in a final sample of 878 cases. Statistical analyses were performed using IBM SPSS Statistics 26.^1^

### Survey content

This section delineates the blocks used for the analysis of tenants’ past choices to move to their current dwellings. Tenant profiles are summarized in the “Appendix” (Tables 7, 8, 9, and 10).

1. *Household composition*, including the socio-demographic characteristics of tenants and their tenancy types. Each respondent’s age, family status (e.g. children at home) and marital status (e.g. divorced) were combined to create household types. Tenants had the option not to answer questions regarding employment, salary and education.
2. *Housing functions*, including tenants’ ideal housing functions at the time of the survey and the functions fulfilled by their past and current dwellings. To avoid misinterpretation, tenants had to evaluate whether each function’s definition described their dwellings independently of its label (5-point Likert scale; 1 = strongly disagree; 5 = strongly agree; see Table 1).
3. *Trigger*, including the trigger event that led to the move and the trigger type associated with the event. A list of trigger events was proposed to the tenants based on (1) a literature review and (2) the results of two previous group discussions during which tenants listed the reasons that pushed them to leave their previous dwelling. In the survey, tenants were given the option to add another answer if none of the proposed corresponded to their choice. The free-form responses were recoded into four new trigger events for a total of 20 events. Tenants directly attributed the chosen events to one of the three trigger types.
4. *Residential satisfaction*, including the tenants’ level of satisfaction with their current and previous dwellings, measured on a 5-point Likert scale (1 = strongly dissatisfied; 5 = strongly satisfied). Satisfaction with the past dwelling was defined as the tenants’ level of satisfaction prior to the trigger event determining the move.

Questions were formulated at the level of the individual in order to capture his/her preferences and understanding of the housing function. However, we acknowledged that because partners in a household attempt to overcome differences between their views, answers could reflect preferences at the scale of the household (Booi & Boterman, 2019).

### Statistical methods

#### Data analysis

To analyse the data, we first performed a descriptive analysis and explored the variables under study. Depending on the variable type, we then ran binary, ordinal and multinomial logistic regression analyses. When the ordinal logistic regression model violated the proportional odds assumption, the multinomial logistic regression was used instead. This was the case for O1 (see Table 2), when tenants’ satisfaction (measured on an ordinal scale) was inputted into the model as categorical variable, meaning that the independent variable measuring the difference between ideal and current housing functions (i.e. ‘gap’) was considered as influencing each category of satisfaction without taking their order into account.^2^

**Table 2:** Operational hypotheses (O), steps of the model, variables and methods

O	Step of the model		Variable		Method
	#	Description	Dependent	Independent	
O1	1	Gap between ideal and current functions is a predictor of satisfaction	Level of satisfaction (*t* + 1)^a^	Gap (*t* + 1)^b^	Multinomial logistic regression
	6				
	7				
O2	2	Trigger effectiveness is related to tenants’ residential satisfaction	Trigger event^c^	Level of satisfaction (*t* − 1)^a^	Multinomial logistic regression
O2	3	Trigger effectiveness is related to the functions of the current dwelling	Trigger event^c^	Current functions (*t* − 1)^a^	Multinomial logistic regression
O3	4	Trigger events determine trigger types	Trigger type^d^	Trigger event^c^	Binary logistic regression
O3	5	Change between past and current functions differs between trigger types	ΔFunctions (*t, t* − 1)^b^	Trigger type^d^	One-way ANOVA
O4	8	Tenants’ characteristics are predictors of ideal functions	Ideal function (*t* + 1)^a^	Socio-demographic characteristics, tenancy type^c^	Ordinal logistic regression

^a^Ordinal variable

^b^Continuous variable, see “Sect. 3.3.2”

^c^Categorical variable

^d^Three dummy variables for three trigger types (0 = no, 1 = yes), see “Sect. 3.3.2”

Furthermore, we used one-way analysis of variance (ANOVA) to compare mean differences of continuous variables between groups defined by a categorical variable (see Step 5 in Table 2).

Table 2 illustrates the variables and methods, the steps of the model to which they refer, and the operational hypotheses they test. The transformations needed to perform the analyses are listed in the following subsection.

#### Data transformation

Data transformation was required to perform the analyses outlined in Table 2. In particular, three new variables were computed:

1. Gap (*t*  +  1) (O1; Step 7)
  For each of the nine housing functions, we computed the variable ‘gap’ as the difference between current and ideal functions at time *t* + 1 (Step 7, proxy for Step 1 and 6). When reality exceeded tenants’ preferences and aspirations (i.e. when the current housing function scored higher than the ideal one), the gap was assigned a value of 0 (i.e. no gap). We formulated it as follows:
  1 $${\text{If}}\;IF_{{ij,t + 1}} \ge CF_{{ij,t + 1}} \;\;{\text{then}}\;\;GF_{{ij,t + 1}} = \left| {CF_{{ij,t + 1}} - IF_{{ij,t + 1}} } \right|,\quad {\text{else}}\;\;GF_{{ij,t + 1}} = 0$$
  where *IF*_*ij,t+*1_ and *CF*_*ij,t+*1_ measure the extent to which a function *j* describes the ideal and current dwelling of a tenant *i* at time *t* + 1 [1 = strongly disagree; 5 = strongly agree], respectively, and *GF*_*ij,t+*1_ measures the gap between residential preferences and reality [0 = perfect match; 4 = largest gap].^3^
2. Trigger type (O3; Step 4)
  From the categorical variable ‘trigger type’ [1 = opportunity, 2 = problem-solving, 3 = radical change], we generated three dummy variables: opportunity [0, 1], problem-solving [0, 1] and radical change [0, 1].
3. ΔFunctions (*t*, *t* − 1) (O3; Step 5)
  To explore the extent to which trigger types adjust the determinants of the decision to select a dwelling, we looked at changes in tenants’ revealed preferences. The variable Δfunctions (*t*, *t* − 1) was calculated as the average absolute difference between the nine current functions at *t* and *t* − 1 (i.e. current and past, respectively):
  2 $$\Delta F_{{t, t - 1}} = {\bar{\text{x}}}\left| {CF_{{ij, t}} - CF_{{ij, t - 1}} } \right|$$
  where CF_*ij,t*_ and CF_*ij,t*−1_ measure whether a function *j* describes the current dwelling of a tenant *j* at time *t* and *t* − 1 [1 = strongly disagree; 5 = strongly agree], respectively, and ΔF_*t*,*t−*1_ measures the change in revealed preferences [min = 0, max = 4].

## Results

### Descriptive analysis

The descriptive statistics for the blocks introduced in the previous section are illustrated in Tables 7, 8, 9, and 10.

The final sample consists of a higher share of cooperative tenants (33.5% from ABZ, 39.5% from SCHL) compared with households renting from the private owner (27% from Mobiliar).^4^ German-speaking tenants (approx. 46%) are similarly but less represented than the French-speaking ones (approx. 54%). Females comprise approximatively 54% of respondents compared with the male proportion of 46%. When grouped into household types, middle-aged couples with children at home constitute the major share of respondents in the cooperatives (24% in ABZ and approx. 18% in SCHL), whereas young couples without children comprise the largest group of tenants renting from Swiss Mobiliar (16.5%). Respondents’ ages range from 22 to 89 years with an average of 51 years (SD = 15.5). Many of the surveyed tenants have a university degree (40% of respondents with a bachelors or masters, plus 5% with PhDs) and are employed either full- or part-time (approximately 71% of respondents).^5^

With regard to residential mobility, the vast majority of the households (95%) has moved in the last 30 years. The most frequently cited reasons for moving are the opportunity to rent another dwelling, an increasing lack of comfort, and household growth (e.g. a new child). Approximately 80% of tenants claim to be satisfied or strongly satisfied with their current dwelling.

Regarding housing functions, housing as a place for ‘production, consumption’ scores the highest for the past (mean = 4.02, SD = 0.82), present (mean = 4.29, SD = 0.69) and ideal dwellings (mean = 4.55, SD = 0.61). Housing as a ‘permanent’ place evinces the greatest increase in importance between past and present dwellings (mean = 0.42, SD = 1.28), while ‘commodity’ exhibits the largest absolute change (increase and decrease; mean = 0.91, SD = 1.02). The greatest difference between current and ideal dwellings is for the function ‘property’ (mean = − 0.92, SD = 1.40), which remains the case when applying the gap formula (mean = 1.02, SD = 1.28; Eq. 1).

### Housing functions in the relocation process: tenants’ satisfaction (O1)

This section examines the applicability of housing functions to understanding households’ residential satisfaction with the goal to determine whether functions play a role in the relocation process (H1; O1).

Table 3 shows the results of the multinomial regression model, whereby the difference between each of the nine current and ideal housing functions at *t* + 1—i.e. the variable ‘gap’—was used as explanatory variable for tenants’ residential satisfaction. The model considers each category of satisfaction against the highest level (‘strongly satisfied’). Its explanatory power is modest (Nagelkerke R^2^ = 0.160) but not unusual (de Groot et al., 2011).

**Table 3:** Multinomial logistic regression of tenants’ residential satisfaction with their dwellings when the gap between each current and ideal housing function increases by one point

	Strongly dissatisfied	Dissatisfied	Neither, nor	Satisfied
*Satisfaction with current dwelling (ref. cat. ‘strongly satisfied’)*				
Intercept	− 2.05*** (0.212)	− 2.51*** (0.216)	− 3.02*** (0.252)	− 0.54*** (0.116)
Property	− 0.3* (0.156) [0.74]	0.00 (0.127) [1.00]	0.12 (0.133) [1.12]	0.13* (0.071) [1.14]
Production, consumption	− 0.17 (0.319) [0.85]	0.44* (0.232) [1.56]	0.33 (0.253) [1.40]	0.44*** (0.149) [1.55]
Impermanence	0.09 (0.201) [1.09]	− 0.11 (0.208) [0.89]	0.00 (0.207) [1.00]	− 0.05 (0.116) [0.95]
Status symbol	− 0.17 (0.375) [0.84]	0.48** (0.243) [1.62]	0.73*** (0.234) [2.07]	0.41** (0.166) [1.5]
Security	0.25 (0.302) [1.28]	0.12 (0.266) [1.13]	− 0.64 (0.394) [0.52]	− 0.14 (0.181) [0.87]
Commodity	− 0.21 (0.318) [0.81]	− 0.47 (0.319) [0.63]	− 0.10 (0.276) [0.90]	− 0.25 (0.162) [0.78]
Self-representation	0.3 (0.226) [1.35]	0.52*** (0.187) [1.68]	0.56*** (0.191) [1.76]	0.04 (0.129) [1.04]
Shelter	0.38 (0.265) [1.46]	0.36 (0.239) [1.43]	0.23 (0.255) [1.26]	0.22 (0.158) [1.24]
Permanence	0.48** (0.232) [1.62]	0.4* (0.208) [1.49]	0.62*** (0.213) [1.86]	0.41*** (0.135) [1.51]
N	878			
Initial − 2LL	1473			
Model − 2LL	1334			
Improvement (Chi^2^)	Chi^2^ = 139.358, df = 36, *p* < 0.001***			
Nagelkerke R^2^	0.160			

Beta coefficients; ***p < 0.01, **p < 0.05, *p < 0.1; (standard error); [odds ratio]

As hypothesized, we observe that for five of the nine functions, the greater the gap between reality and preferences, the greater the odds of not being strongly satisfied with the current residential condition. More specifically, the more tenants imagine their ideal dwelling as a place where they belong (i.e. ‘permanence’) or as a place for the ‘self’ (i.e. ‘status symbol’ and ‘self-representation’), the more likely they are to be dissatisfied or even strongly dissatisfied when their current dwelling doesn’t fulfil that function. However, the findings also show that gap variables are not consistently significant across categories of residential satisfaction; for instance, a one-unit increase in gap for the function ‘self-representation’ does not discriminate between ‘strongly satisfied’ and ‘satisfied’.

Two predictors evince unexpected results: ‘property’ and ‘production, consumption’. Regarding the first, as opposed to the other regression coefficients, results show that the greater the difference between ideal and current functions, the lower the chances of being strongly dissatisfied (OR = 0.74; 10% sig. level). Regarding the second, it can be observed that for a one-unit increase in the gap between reality and preferences, the odds of being satisfied rather than strongly satisfied increase by 55%. Considering that a place for ‘production, consumption’ refers to the performance of daily activities (e.g. eating, laundering), the function is expected to be determinant in discriminating a lower level of satisfaction from a higher one rather than a high level from the highest.

### The influence of housing functions on the determinants of the decision to move (O2)

Having clarified the link between housing functions and residential satisfaction, this section examines their relationship with the determinants of the decision to move: the triggers. Triggers can generate from gradual changes (e.g. decrease in comfort, increase in stress), or sudden ones (e.g. a divorce); they can arise from the tenant’s life course trajectory (e.g. new job location), or they can be caused by the management and dynamics of the housing stock (e.g. a demolition).

Table 11 in the “Appendix” displays the results of the multinomial logistic regression model, which estimates the effects of the level of satisfaction and the nine housing functions on the effectiveness of a trigger event. The explanatory power of the logistic regression model with all predictors (Nagelkerke R^2^ = 0.306) is improved compared to the model limiting its predictors to the level of satisfaction (Nagelkerke R^2^ = 0.057) or to the functions (Nagelkerke R^2^ = 0.281).^6^ The model considers each event against the trigger ‘increasing lack of comfort’.

#### Trigger events and residential satisfaction

Table 11 shows that, overall, the more tenants are satisfied with their dwelling, the less likely they are to move due to the reference category ‘lack of comfort’. Our interest is focused more specifically on ranking the odds, which shows the power of each trigger event against the level of satisfaction (Table 4).

**Table 4:** Ranked overview of odds ratios of significant predictors from the multinomial logistic regression analysis of moving due to a trigger event when the level of satisfaction increases by one point

Trigger event	
Change in life-location	3.72**
Forced to move	1.88***
Rental contract expiration	1.57*
New job location	1.56***
Need for a radical change	1.55**
Move with partner	1.44***
Rent too high	1.43**
Dwelling too small	1.42**
Children leaving home	1.40**
Need for autonomy	1.39*
Household growth	1.34**
Divorce, separation, loss of partner	1.32**
Opportunity to rent	1.29**

***p < 0.01, **p < 0.05, *p < 0.1

The higher the level of satisfaction, the more likely it is that the trigger events resulting in a move are problems generated either by the housing stock (e.g. a forced move, a rental contract expiration) or the tenant’s educational or occupational career; for instance, tenants are nearly four times more likely to move because of a change in life-location than a lack of comfort when the level of satisfaction increases by one point (OR = 3.72). Changes in household career—such as an explicit need for radical change, a move with the partner or the shrinking and growing of the household—are also found to be from 32% to 55% more likely to be effective with a higher residential satisfaction.

Compared to a lack of comfort, the opportunity to rent another dwelling or be accepted in a cooperative displays the lowest odds of moving with a satisfaction increase of one point (OR = 1.29). To consider and catch an opportunity, the tenant is indeed expected to have a lower level of satisfaction.

#### Trigger events and housing functions

Table 11 indicates that the trigger event leading to the move significantly depends on the function fulfilled by the dwelling.

When the dwelling is considered a place to belong (i.e. permanence), households are more likely to move due to relevant changes in their life-course (e.g. leaving the parent(s)’ home, OR = 8.35; children leaving home, OR = 2.20) or the imposed circumstances (i.e. forced to move, OR = 2.32; dwelling too small, OR = 1.52) rather than a decrease in comfort. The same is the case when the dwelling meets the needs of a specific life phase (i.e. impermanence); for instance, when this function is perceived as 1-point stronger in one’s dwelling, the odds of moving due to retirement increase by a factor of 2.11. Similarly, tenants who consider their dwelling a place for ‘self-representation’ or a ‘status symbol’ are overall more reticent to move unless an event such a divorce (which supposedly imposes a change in status and the self) impels it (self-representation OR = 1.52; status symbol OR = 1.32, 10% sig. level). Lastly, results show that the ‘homely home’ or ‘shelter’ is more likely to be left due to a move with the partner rather than a decrease in comfort (i.e. rebuilding a shared shelter; OR = 1.47).

Compared to these results, dwellings labelled as ‘properties’, places for ‘production, consumption’ or ‘commodities’ evince the opposite regression coefficients; tenants living in such dwellings are more likely to move due to a lack of comfort than other trigger events. Among these, only the function ‘commodity’ indicates an exception; when the dwelling is considered a temporary or convenient place, a raise in salary can be the perfect opportunity for change (OR = 2.24, 10% sig. level).

### Change in preferences following a trigger (O3)

The findings of the previous section confirmed the hypothesis that for most of the functions, the level of satisfaction with the dwelling where tenants reside and the housing functions that it fulfils indicate the extent to which a trigger event is effective. This section tests the hypothesis that these triggers can be categorized into types with varying impacts on adjusting tenants’ preferences for the new dwelling (H3; O3).

#### From trigger events to trigger types

To organize the variety of determinants to move, the survey asked tenants to assign the event that impelled them to move to one of the three proposed types: opportunity, problem-solving, or radical change. Table 5 displays an overview of the significant predictors of each trigger type resulting from three binary logistic regressions (for the full table, see Table 12 in the “Appendix”).

**Table 5:** Ranked overview of odds ratios of significant trigger events predicting each type of trigger leading to the move

Opportunity^a^		Problem-solving^b^		Radical change^c^	
Raise in salary	7.00**	Rental contract expiration	40.22***	Leaving parent(s)’ home	67.50***
Opportunity to rent	6.76***	Interpersonal problems	19.80***	Divorce, separation, loss of partner	20.53***
Divorce, separation, loss of partner	0.05***	Rent too high	13.29***	Need for a radical change	16.07***
Rental contract expiration	0.23*	Change in life-location	12.38***	Move with partner	13.59***
New job location	0.39**	Lack of space	9.72***	New job location	12.92***
		Family (ageing, children)	9.90***	Need for autonomy	10.91***
		Forced move	8.57***	New child or household growth	9.03***
		Increasing lack of comfort	7.46***	Children leaving home	6.30**
		Accessibility	6.88***	Retirement	5.36*
		Divorce, separation, loss of partner	3.90***		
		New job location	2.78**		

***p < 0.01, **p < 0.05, *p < 0.1

^a^The reference category is ‘Forced move’

^b^The reference category is ‘Opportunity to rent’

^c^The reference category is ‘Rental contract expiration’

Firstly, results show that whether a trigger is perceived as a problem is more likely to depend on events that are ‘external’ to the household. The most important problem to solve is the rental contract expiration (OR = 40.22), followed by interpersonal problems with neighbours or flatmates (OR = 19.80). Additional predictors include issues related to the rented dwelling (e.g. a rent too high, OR = 13.29; lack of accessibility, OR = 6.88) and educational or occupational career events (e.g. the ‘family’—meaning for instance the need to move closer to locations relevant for children’s education, OR = 9.90; a change in life-location, OR = 12.38; a new job location, OR = 2.78, 10% sig. level).

Secondly, changes exclusively related to life course trajectories are relevant predictors of ‘radical change’: along with the explicit need for a radical change (OR = 16.07), a divorce (OR = 20.53), a move with a partner (OR = 13.59), a new job location (OR = 12.92) and households’ growth (OR = 9.03) are significantly related to this typology. The strongest predictor is leaving the parent(s)’ home, which when compared to the reference category ‘rental contract expiration’ increases the odds of considering the move as a radical change by a factor of 67.

Lastly, we can observe that the odds to move for an opportunity decrease between 95 and 61% when the trigger pushing the move is a divorce, a rental contract expiration or a new job location. This finding indicates a clear distinction between opportunity and the two other triggers; however, there is a less stark difference between problem-solving and radical change, which can encompass both the loss of the partner or a new job location (although the odds are significantly higher for the third trigger type).

#### Change in housing functions with trigger types

The results of the one-way ANOVA for the full sample of respondents indicate that the trigger type significantly influences the extent to which housing functions change between the former dwelling (i.e. current at time* t* − 1) and the current residence at time *t* (ΔFunctions; Table 6). However, the trigger type explains only 1.2% of the spread of this change around the overall mean (adjusted R^2^), and the effect size is rather weak (*f* = 0.1; Cohen, 1992).

**Table 6:** One-way ANOVAs between trigger types on the mean change in functions between current dwellings at *t* and *t* − 1 for the full sample and the ‘strongly satisfied’ subsample

	N	Mean	SD		SS	df	MS	*F*	Sig.	η_p_^2^
*Full sample*										
OP	323	0.62	0.45	Between groups	3.11	2	1.556	6.317	0.002***	0.014
PS	217	0.71	0.53							
RC	338	0.76	0.52	Within groups	215.56	875	0.246			
Tot	878	0.70	0.50	Tot	218.67	877				
*Subsample ‘strongly satisfied’*										
OP	55	0.46	0.36	Between groups	4.77	2	2.386	9.079	0.000***	0.088
PS	51	0.79	0.61							
RC	86	0.82	0.53	Within groups	49.67	189	0.263			
Tot	192	0.71	0.53	Tot	54.45	191				

Full sample R^2^ = 0.014; Adjusted R^2^ = 0.012

Subsample R^2^ = 0.088; Adjusted R^2^ = 0.078

*SS* sum of squares, *MS* mean of squares, *Tot* total, *OP* opportunity, *PS* problem-solving, *RC* radical change

***p < 0.01, **p < 0.05, *p < 0.1

Additionally, post-hoc tests with Bonferroni correction reveal that the change in functions following the trigger type ‘problem-solving’ does not significantly differ from the two other types, whereas ‘opportunity’ and ‘radical change’ do (mean difference 0.14; *p* < 0.01; Table 13 in the “Appendix”). This result indicates that contrarily to H3, a problem to solve can lead to both a strong and a weak change in housing functions. However, it also confirms that tenants who moved due to a radical change in their lives rather than an opportunity chose dwellings with significantly different functions than those of their previous residence.

To further investigate changes in preferences in relation to trigger types, Table 6 also displays the results of the one-way ANOVA for those respondents with the highest level of satisfaction prior to the trigger (5 points over 5). For this population, the gap between current and ideal housing functions before the move is supposed to be minimum, and no adjustment in housing functions for the new dwelling is therefore expected following the occurrence of an opportunity or a problem to solve.

Compared with the full sample, results for this subset show a moderate improvement—adjusted R^2^ = 0.078, medium effect size (*f* = 0.31; Cohen, 1992). As was the case for the full sample, post-hoc tests with Bonferroni correction indicate that ΔFunctions following an opportunity significantly differs from ΔFunctions following a radical change with a mean difference of 0.36 points (*p* < 0.01). However, contrarily to the hypothesis (H3), the category ‘problem-solving’ also elicits a significantly greater change in function compared with ‘opportunity’ (+ 0.33 points, *p* < 0.01).

### Tenants’ characteristics and ideal housing functions (O4)

A variety of household characteristics play a role in the decision to move and where to move. Table 14 in the “Appendix” displays the result of the ordinal logistic regressions, whereby household type, employment status, salary, education level and tenancy type (i.e. housing owner) are used as explanatory variables of tenants’ preference for each housing function—i.e. ideal function at *t* + 1. According to the test of parallel lines, or the proportional odds assumption, five of the nine models are equal across outcome levels (chi-square > 0.05) and are therefore included in the table. To facilitate their presentation, we illustrate the results of the models in four subsections.

#### Property

The first model displays the largest range of significant predictors.

We firstly observe that singles (18–64 years) and young couples (18–34 years) are between two to almost six times more likely to aspire to have a place that ‘belongs’ to them compared with middle-aged tenants with children. The relative probability of considering such a place as ideal decreases by nearly 60% for couples above retirement age (10% sig. level; Table 14). Secondly, compared with a university degree, holding a high school diploma also indicates a lower likelihood to wish for a ‘property’ (OR = 0.49). Lastly, this likelihood is 51% greater for households renting from the private sector (i.e. Swiss Mobiliar) compared with those in the SCHL cooperative.

Employment rate and salary are also significant explanatory variables of the function ‘property’. On the one hand, being unemployed decreases the likelihood of desiring this function for one’s dwelling (OR = 0.49); on the other hand, and surprisingly, the second lowest category of salary decreases it compared to the first (OR = 0.60). It must be considered that the variable ‘salary’ accounts for the sum of the salaries of all household members, which is expected to be lower for one-person households—this is particularly pertinent given that the category ‘single’ is a significant predictor of this function.

To summarize, the profile of tenants considering the housing function ‘property’ as ideal can be outlined as renters having just started their housing and household careers: single or young couples, tenants with lower salaries, a higher level of education, working full-time, and renting from a private owner rather than being part of a cooperative system.

#### Status symbol

Young singles and full-time workers are also attracted by the function ‘status symbol’. Results show that the likelihood of considering housing as a credential for esteem most strongly increases when the tenant is a young single (18–34 years, OR = 2.08), and working full time (working part-time decreases the odds of considering the status symbol 1-point more ‘ideal’ by 78%). Interestingly, significant predictors include renting from ABZ (OR = 1.58) and Mobiliar (OR = 1.40), whose dwellings are predominantly located in the Swiss-German part of Switzerland.

However, it must be noted that the explanatory power of this model is weak (R^2^ = 0.083), which is also the case for the next model: impermanence (R^2^ = 0.085).

#### Impermanence and shelter

A dwelling ‘free from tradition or memory’ (i.e. impermanent) is the ideal place for ‘lonely’, middle-aged tenants who are divorced or widowed and do not have children (OR = 2.42). In addition, Table 14 shows that renting from a private owner rather than a cooperative increases the likelihood of desiring a place that merely reflects current needs by 51%.

Single (OR = 1.46) or divorced middle-aged tenants (OR = 2.48) are also in search of a ‘shelter’; however, this function is most strongly desired by young couples (with children, OR = 3.81; without children OR = 3.90). In addition, when growing old (i.e. middle age), couples are approx. 60% less likely to desire the ‘shelter’ function when their children are gone than when they are still living in the dwelling. In summary, ‘shelter’ fits well to a broad range of tenants, such as young couples, families of mid- and younger age, and lonely tenants. Again, the predominant location in the Swiss-German part of Switzerland (i.e. ABZ, OR = 2.47; Mobiliar, OR = 1.44, 10% sig. level) is a significant predictor of this function.

#### Permanence

In addition to ‘property’ and ‘shelter’, young couples without children also long for ‘permanence’ (OR = 2.18), or a place to feel rooted, which is consistent with considering this household type as just starting its housing career and therefore imagining the dwelling as its own stable and cosy place. As is the case for ‘shelter’, the likelihood of considering such place as ideal decreases when the children leave the nest (OR = 0.37).

Moreover, we point to the finding that employment is another significant explanatory variable for this model. More specifically, the odds of considering the ideal dwelling as a permanent place increase by a factor of 3.31 when the tenant spends more time at home, i.e. is a housewife or househusband.

## Discussion

In this paper, we investigated the role played by housing functions in the residential mobility of the tenants of a real estate owner and two of the largest cooperatives in Switzerland. Based on prior qualitative research, we introduced a multi-step theoretical model of tenants’ decision to relocate (Fig. 2) and then explored its linkages by means of empirical analyses of survey data.

In the following subsections, we discuss the results of this study along four lines: first, we present a synthesis of the findings and their theoretical contribution; second, we illustrate potential implications for practice; third, we discuss the study’s limitations; lastly, we identify promising avenues for future research.

### Research findings in perspective: disentangling systems complexity

The first hypothesis scrutinized in this study was that housing functions can be used as proxies for residential attributes (housing, neighbourhood, location) to understand households’ satisfaction with their dwellings and thus are relevant for unravelling the decision to move and the selection process (H1).

Results have shown that, in most cases, residential satisfaction is more likely to increase with a decreasing gap between the housing functions of the ideal and current dwelling. However, we also observed that these findings are not consistently significant across categories of satisfaction. More specifically, the fulfilment of a housing function was found to make large or little-to-no difference to tenants’ residential satisfaction—e.g., for certain functions the gap was a significant predictor of the jump between a strong dissatisfaction to a strong satisfaction or vice-versa, for others of the jump between ‘neither nor’ to ‘strongly satisfied’ or vice-versa. In agreement with recent studies that have disproved the commonly explored existence of a linear relation between satisfaction and gap (see e.g. Jiang et al., 2020), our choice of a multinomial regression model pointed to the different influences that housing functions can have on rather than across categories of satisfaction. Also, our findings contribute to the research of the many scholars who, since the seminal work of Rossi (1955), have attempted to disentangle the complex links between residential satisfaction and the determinants of residential mobility (see, for instance, the conceptual model proposed by Marans, 1976). In particular, the existence of a *direct* or *‘mechanistic’* relationship between the residential environment and household satisfaction has often been questioned (Lawrence, 1987; Michelson, 1980), arguing that the latter can vary within and between households who subjectively interpret and assess the objective characteristics of the former (i.e. (dis)amenities), depending on a variety of factors (expectations, reference groups, subjective beliefs; Cook & Bruin, 1994; Diaz-Serrano & Stoyanova, 2010; Galster, 1987; Galster & Hesser, 1981; Jansen, 2014; Jiang et al., 2020; Marans, 1976). By introducing the functions as mediators between the human and material subsystems and thereby accounting for both tenants’ preferences and dwelling forms, this study does not advocate for the existence of a *direct* relationship between satisfaction and dwelling but rather an *indirect* and systemic one. This conceptualization makes it possible to overcome the limitations encountered in other authors’ empirical analyses, and in particular the aforementioned subjective ways but also the complex combinations in which dwellings features affect residential satisfaction—i.e. the correlations between and within categories of residential attributes (dwelling, neighbourhood, location) or the different effects that each of these categories has been found to exert on residential satisfaction (Jiang et al., 2017; Molin et al., 1996; Wong, 2002). In other words, our results demonstrated that the notion of housing function can offer a shortcut to link residential satisfaction to the objective and subjective characteristics of the environment and of its residents while accounting for the system’s complexity.

The findings of H1 are of relevance given that residential satisfaction plays a role in the decision to move and the formulation of preferences for the new dwelling. When looking at the former, we found that housing functions both directly and indirectly influence the extent to which tenants are likely to move following an event (e.g. a new child; H2). More specifically, we observed that the level of residential satisfaction (which itself is influenced by the size of the gap between ideal and current functions) and the function that the dwelling fulfils are significant explanatory variables of the event triggering the move. Building on the seminal work of Speare (1974), most scholars have examined the direct and indirect relations between households’ mobility, residential satisfaction, housing features and socio-demographic characteristics (for an overview, see Jiang et al., 2017). Our findings contribute to this body of literature by focusing on the effects that satisfaction and housing functions (and therefore housing and residents’ characteristics) have on the *triggers* of the relocation process, rather than on the intention and actual behaviour.

Similar research was undertaken by Wong (2002), whose results showed that the triggers to move have ‘unequal correlations’ with households’ level of satisfaction (p. 227). By grouping triggers into types (i.e. opportunities, problems to solve, and radical changes), our model extends her results one step further. More specifically, when comparing Table 4 with Table 5 and confirming former exploratory findings (Pagani & Binder, 2021), we observe that the trigger events that are the most effective with an increasing level of satisfaction are often the predictors of the imposed triggers or ‘forced’ moves (Clark & Onaka, 1983), i.e. ‘radical change’ and ‘problem-solving’.

When looking at the formulation of residential preferences and by further exploring the systems interrelations between housing functions and triggers, our findings demonstrate that trigger types differently arbitrate the change in function for the new dwelling (H3). More specifically, despite the weak-to-medium effect size, a radical change was found to most strongly affect tenants’ preferences in terms of housing functions. This finding first supports the argument that relocations are instrumental to goals, which can change during the household’s life course (Coolen et al., 2002; Mulder & Hooimeijer, 1999); second, it corroborates H1 by showing that housing functions are a constituent element of these ‘goals’.

Based on this observation and on the body of literature introduced in this paper, households’ characteristics were expected to influence housing functions in multiple ways (H4). Our regression models confirm that household type (marital status, age and children) is a significant explanatory variable for five of the nine ideal housing functions. The findings also illustrate the diversity of ideal dwellings resulting from combinations of different *careers* (e.g. educational, occupational; Mulder & Hooimeijer, 1999), including the type of tenancy.

As outlined in this section, our findings contribute to the body of literature on residential mobility by illustrating the potential of introducing the notion of housing functions for disentangling the complexity of the human–environment system under study. More specifically, our results suggest that the functions orchestrate the factors leading to the moves of Swiss tenants (i.e. triggers, satisfaction and preferences).

### Relevance for practice

In agreement with several scholars, this study argued that a better understanding of the relocation process and its determinants can play a key role in fostering the provision of adequate, appropriate, and quality housing—i.e. dwellings that support and meet the culture, values and needs of households for which those are intended (see for instance Clark et al., 2006; Franklin, 2001; Kahlmeier et al., 2001; Lawrence, 2004; Molin et al., 1996; Rapoport, 1977). Due to the housing system’s complexity, disagreement between housing providers (i.e. owners, practitioners, policy makers) and users (i.e. residents) on what constitutes residential quality persists (Diaz-Serrano & Stoyanova, 2010; Franklin, 2001; Jansen, 2014; Lawrence, 2009, 2021a; Marans, 1976), which can have several implications. For instance, the difficulty in understanding the links between objective and subjective assessments of the residential environment can undermine the success of housing developments or neighbourhoods—when the housing situation is dissatisfactory, the residents consider housing alternatives (Cook & Bruin, 1994; Kwon & Beamish, 2013; Lawrence, 2009); also, dissatisfaction has been demonstrated to have repercussions beyond households’ relocation, and especially to impact residents’ health and well-being (Clark & Kearns, 2012; Jansen, 2014; Kahlmeier et al., 2001; Rolfe et al., 2020).

For these reasons, it has long been argued that plans and programs related to providing or improving housing quality must include final users in the discussion (Lawrence, 2021a). However, participatory approaches might be insufficient if tools to disentangle the system’s complexity and foster the integration of the multiple stakeholders’ perspectives are not available. Therefore, based on the results presented in our study, practitioners should consider the added value of adopting a systems perspective and using the notion of housing functions for accounting for the relative value that different residents’ groups attach to specific dwelling, neighbourhood and location features while ensuring a comprehensive assessment and provision of the many ‘interrelated purposes that impinge upon the quality of the [residential] environment’ (Lawrence, 1995, p. 1663).

### Limitations

While the multi-step model proposed in this study offers a new take on the conceptualization of the residential mobility process, several limitations must be acknowledged. Mainly, the results of the analyses were not consistently significant for the nine housing functions: on the one hand, they were sensitive to the chosen regression models (i.e. ordinal, multinomial; e.g. Table 14); on the other hand, they were influenced by the choice of the variable to investigate. Below, we discuss the effects of models and variables on our results.

#### Gap and satisfaction

Looking at the data of Table 3, four of the nine functions are not significant in the regression model. When comparing it with Table 8, it can be observed that ‘commodity’, ‘impermanence’ and ‘security’ are on average fulfilled more than tenants desire (see variable ΔCurrent-Ideal). This result shows the limitation of the formula chosen to compute the gap between reality and preferences, which considers only the *lack* of a dwelling function as a predictor of residential satisfaction, regardless of its *abundance*. Rather, more complex models have assumed the existence of an ideal point, whereby satisfaction decreases if reality deviates from aspirations in both directions (see e.g. Jiang et al., 2017, 2020); in other words, a function might also be perceived as *undesirable* or conflictual and thereby negatively affect tenants’ level of satisfaction (e.g. a dwelling ‘free from tradition and memory’ *versus* the need for a place ‘where I feel rooted’). In addition, to account for residents’ different sensitivities to under- and outperformance of a preference, Jiang and colleagues (2020) proposed non-linear asymmetric gap models which consider that the same gap might not always lead to the same level of dissatisfaction. Also, beside the generally used difference formulation, the authors computed the size of the gap as a relative difference, i.e. dependent on how great the level of aspiration is.

Aside from the way variables were computed, the predominance of moderately and totally satisfied tenants in the sample of respondents is a relevant limitation (see Table 10); this bias or dissonance is common in other studies, and derive from a tendency of evaluating a past decision positively (Jansen, 2014; Kahlmeier et al., 2001; Marans, 1976).

In sum, residential satisfaction is a complex notion that has been conceptualized, measured, and calculated in manifold ways and is subjected to several biases. In this study, the way the dependent and independent variables were computed revealed several limitations which could be overcome by more methodologically advanced gap models.

#### Trigger types

Asking tenants to assign the trigger events to one of the three proposed types aimed at validating the typology of triggers proposed in the Pagani and Binder’s (2021) qualitative study. However, while observing the richness of events that can be categorized as problems to solve or radical changes, we also faced the issue of having the same event categorized in both types.

More specifically, a closer examination of Tables 5 and 11 shows that the links between functions, trigger events and trigger types remain unclear. For instance, the function ‘property’ was found on the one hand to increase the likelihood of moving due to trigger events categorized as ‘radical changes’ or ‘problems to solve’ and on the other hand to decrease the likelihood of moving due to a ‘decrease in comfort’, which tenants also classified as a problem to solve. Another example is Table 6, where an update in housing functions—which was only expected for the category ‘radical change’—was observed following the trigger ‘problem-solving’, a result that could also be explained by the above-mentioned overlapping of types per event.

These unclear relationships potentially suggest the existence of sub-categories of the three trigger types depending on the triggering ‘power’ of each event in the type, meaning the level of satisfaction at which they are effective.

#### ΔFunctions and trigger types

The choice to compare changes in current housing functions (i.e. between past and present dwelling) to observe the effects of triggers on residential preferences should also be discussed. One could argue that this approach is correct only if the current housing function (i.e. revealed preferences) corresponds to the ideal one (i.e. stated preferences). If not, the tenant would take advantage of any trigger type to choose a dwelling that better matches its ideal functions (Pagani & Binder, 2021). At time *t*, the result of the move would evince an update in current functions, which would not correspond to an update in the ideal ones.

In agreement with this argument, results for the subsample who moved with a high level of satisfaction (i.e. with current and ideal functions matching; see H1) showed improved results compared with the full sample (Table 6). However, contrary to H3, the trigger ‘problem-solving’ brought about an unexpected and significantly greater change than the trigger ‘opportunity’. Possible explanations for this result emerge when considering the context, as illustrated in the next subsection.

#### Beyond variables: the relevance of the context

The extraordinarily low vacancy rate in Switzerland cannot be overlooked when investigating tenants’ residential choices. Although encompassed by the trigger events, the influence of micro- and macro-level contexts was not thoroughly accounted for in the variables chosen for our analysis of preferences. In fact, analysing the stated and revealed preferences through ideal and current housing functions did not account for the adjustments of the criteria to *what is possible* (Timmermans et al., 1994; van Ham, 2012); elements such as income or the availability of dwellings on the market can make preferences and final selections deviate from ideal housing functions. This is clear in Sect. 4.5, where salary and education were found in most cases not to be good predictors of ideal housing functions. This argument is also key for our interpretation of Sect. 4.4, whereby the trigger ‘problem-solving’ was found to bring about an unexpected change in function; considering time constraints (i.e. contract expiration), a compromise between the dwellings available on the market and the ideal one is often needed, thereby potentially resulting in a change in function. Further, the results presented in Sect. 4.1 show that fulfilment of the function ‘production, consumption’—which encompasses basic activities such as laundering or social activities such as companionship—is relevant but not sufficiently critical to discriminate a low from a high level of satisfaction; this finding should be further investigated in relation to the Swiss economic and sociocultural context (e.g. wealth, interpersonal relationships).

Previous studies have accounted for resources and restrictions (e.g. household salary), and opportunities and constraints (e.g. vacant dwellings) when investigating the decision process by adopting the so-called ‘three-stages approach’ (Mulder, 1996; Mulder & Hooimeijer, 1999). Following this approach, a new function could be introduced: the *desired* function. As the *ideal* function is only dependent on a household’s trajectories, the *desired* function would correspond to the adaptation of the ideal one to resources and restrictions, and the *current* function to the adaptation of the desired one to opportunities and constraints. These three types of functions would more specifically account for the trade-off between the multiple determinants that arise from, for example, lifestyle and individual resources (Thomas & Pattaroni, 2012) and the re-evaluation of preferences in the search process (Brown & Moore, 1970).

### Future research

Based on the limitations illustrated above, it becomes clear that further research is needed. Firstly, the role of housing functions in the selection process should be more closely considered by (1) focusing on the readjustment of the ideal housing function(s) to the desired one(s) following a trigger and of the latter to the current one(s) for the final selection; (2) critically analysing the contribution of the three types of functions to households’ satisfaction with and selection of a dwelling; and (3) exploring the potential to use previously-identified explanatory variables for tenants such as age, size of household and rent as predictors of the desired function (Clark & Dieleman, 1996). In particular, further studies of the relationship between housing functions and resident satisfaction could benefit from the substantial methodological advances in the field, e.g. the use of non-linear models (Jiang et al., 2020).

Secondly, while our study investigated tenants’ past move—where the intention to move corresponds to actual residential mobility—new insights could be gained by examining unsuccessful relocations (Coulter, 2013); in this context, the factors preventing relocation identified in the large amount of research based on the stress-resistance models could be explored in relationship to housing function and trigger types (i.e. the monetary and non-monetary costs of moving; see Brown & Moore, 1970; Clark & Onaka, 1983; Goodman, 1976; Mulder, 1996; Phipps, 1989; Phipps & Carter, 1978; Wolpert, 1965).

Thirdly, this paper presented the results of quantitative research conducted in the framework of the Swiss rental market which are country- and tenure-specific; considering the relevance of the context for the present and future studies, the *tenancy* type and the influence it has on tenants’ decisions could also benefit from further research (e.g. due to occupancy rules, a reduction in household size can result in a ‘forced move’ for cooperative tenants). Furthermore, while the notion of housing functions allowed us to consider and have a better understanding of the interrelationships at play in the housing system (i.e. objective and subjective assessments of housing quality, changes in residential preferences, residential satisfaction, etc.), additional qualitative and quantitative research could be conducted to explore the functions’ potential material manifestations in the Swiss context for different inhabitants’ groups.

Lastly, for our results to appeal to decision-makers and practitioners, and thereby reduce the so-called ‘applicability gap’ (Lawrence, 2021b), the proposed model of residential mobility should be explicitly integrated with context dynamics, i.e. opportunities and constraints generated by the housing market. Since a systems perspective was adopted, an agent-based model (ABM) can be utilized for this purpose. The goal of an ABM is to observe the parallel actions of components and their interaction, thereby discovering emergent properties from a bottom-up perspective (Nikolic & Ghorbani, 2011). Implementing an ABM would make it possible to simulate the system outlined in this paper (i.e. tenants’ residential relocation process) and integrate it with housing stock dynamics (i.e. construction, demolition, renovation). By accounting for the material components of housing and stakeholders’ goals, priorities and values, the model would contribute to a greater understanding of the behaviour of such a complex human–environment system and thereby make it possible to observe otherwise-unpredictable reciprocal effects between residential preferences and dwellings.

## Conclusion

This study investigated the role of housing functions as orchestrators of tenants’ residential mobility in Switzerland. We operationalized previous qualitative work in a multi-step model and explored it by means of survey data. The survey targeted the tenants of a Swiss real estate owner and of two of the country’s largest cooperatives.

Our analyses showed that tenants’ residential satisfaction is more likely to increase when the gap between ideal housing functions and those actually fulfilled by the current dwelling decreases. As residential satisfaction is relevant both in the decision to move and the formulation of preferences, there is a potential to use housing functions to understand the relocation process*.* Secondly, we found that these functions both directly and indirectly influence the likelihood of an event triggering a move; the effectiveness of such triggers was observed to depend on the satisfaction prior to the event (e.g. a rental contract expiration is more powerful than an opportunity to rent a dwelling elsewhere) and the function fulfilled by the dwelling (e.g. a place for ‘self-representation’ being left for events such as a divorce). Additionally, we found that trigger events can be grouped into types (i.e. opportunities, problems to solve and radical changes), which were found to influence the change in housing function(s) before and after the move to a certain degree. This change is further explained by the significance of socio-demographic data and tenancy type as predictors of *ideal* functions, as these data are updated after radical changes (e.g. leaving the parent(s)’ home).

Finally, the use of current and ideal functions was found to be key for depicting Swiss tenants’ residential preferences. However, this paper discussed several limitations in the models and variables chosen for the analysis and highlighted the need for a better integration of micro- and macro-contextual elements in the analysis of preferences. In this framework, our study could benefit from the integration of a new variable: the *desired* function. This variable would account for the adjustment of the ideal functions to tenants’ resources and restrictions and then be further adapted to the available housing supply, thereby resulting in the selection of the most satisfactory current function.

Having a greater understanding of the complex human–environment interactions in the housing system is key for research and practice that targets the supply of adequate and quality housing, and thereby residents’ health and well-being. With this purpose, the findings of this study could be simulated by means of an ABM that integrates the proposed model with supply-side constraints and opportunities.

## Figures and Tables

**Table 11:**
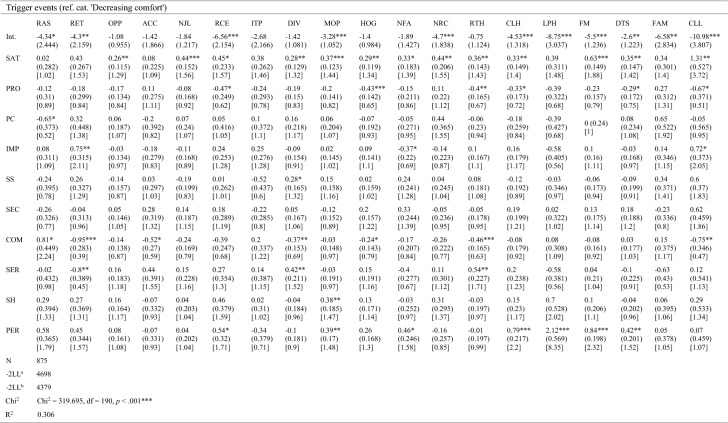
Multinomial logistic regression of moving for a trigger event compared to the reference category ‘increasing lack of comfort’ when the level of satisfaction prior to the trigger increases by one point

Dependent variables: *RAS* raise in salary, *RET* retirement, *OPP* opportunity to rent, *ACC* accessibility, *NJL* new job location, *RCE* rental contract expiration, *ITP* interpersonal problems with neighbours, flatmates; *DIV* divorce, separation, loss of partner; *MOP* move with partner, *HOG* household growth, *NFA* need for autonomy, *NRC* need for a radical change, *RTH* rent too high, *CLH* children leaving home, *LPH* leaving parent(s)’ home, *FM* forced move, *DTS* dwelling too small, *FAM* family (ageing, children), *CLL* change in life-location

Predictors: *Int* intercept, *SAT* level of satisfaction at time *t* − 1, *PRO* property, *PC* production, consumption; *IMP* impermanence, S*S* status symbol, *SEC* security, *COM* commodity, *SER* self-representation, *SH* shelter, *PER* permanence

Beta coefficients; ***p < 0.01, **p < 0.05, *p < 0.1; (standard error); [odds ratio]

− 2LL^a^ initial − 1 log likelihood; − 2LL^b^ model − 2 log likelihood; Nagelkerke R^2^

## Data Availability

The datasets analysed during the current study will be made available in a public repository upon completion of the research project ‘Shrinking Housing’s Environmental Footprint (SHEF)’.

## Appendix

See Tables 7, 8, 9, 10, 11, 12, 13, and 14.

**Table 7:** Respondent profiles for block 1: household composition

Variable [0, 1]	Full sample		ABZ		SCHL		Mobiliar	
	N	%	N	%	N	%	N	%
Sex	877	100	294	100	346	100	237	100
Female	472	53.8	171	58.2	187	54	114	48.1
Male	405	46.2	123	41.8	159	46	123	51.9
Household type	870	100	292	100	344	100	234	100
Young single	43	4.9	8	2.7	17	4.9	18	7.7
Young couples without children	73	8.4	10	3.4	24	7.0	39	16.7
Young couples with children	26	3.0	9	3.1	8	2.3	9	3.8
Middle-aged single	88	10.1	24	8.2	37	10.8	27	11.5
Middle-aged couples without children	66	7.6	18	6.2	20	5.8	28	12.0
Middle-aged alone without children	59	6.8	23	7.9	27	7.8	9	3.8
Middle-aged couple with children living at home	163	18.7	70	24.0	61	17.7	32	13.7
Middle-aged couple with children not living at home	46	5.3	19	6.5	20	5.8	7	3.0
Middle-aged alone with children living at home	50	5.7	20	6.8	18	5.2	12	5.1
Middle-aged alone with children not living at home	38	4.4	17	5.8	16	4.7	5	2.1
Other middle-aged couples	19	2.2	6	2.1	7	2.0	6	2.6
Older couple	95	10.9	28	9.6	43	12.5	24	10.3
Older alone	104	12.0	40	13.7	46	13.4	18	7.7
Employment	848	100	286	100	330	100	232	100
Full-time 80–100%	430	50.7	121	42.3	153	46.4	156	67.2
Part-time < 80%	171	20.2	86	30.1	61	18.5	24	10.3
Housewife/househusband	17	2.0	6	2.1	6	1.8	5	2.2
Student or apprenticeship	8	0.9	4	1.4	1	0.3	3	1.3
Unemployed	18	2.1	1	0.3	14	4.2	3	1.3
Retired	204	24.1	68	23.8	95	28.8	41	17.7
Salary	701	100	235	100	280	100	186	100
Less than 60,000 CHF/year	229	32.7	90	38.3	111	39.6	28	15.1
60.0001–88,000 CHF/year	211	30.1	79	33.6	89	31.8	43	23.1
88,001–120,000 CHF/year	149	21.3	42	17.9	48	17.1	59	31.7
120,001–164,999 CHF/year	67	9.6	14	6.0	20	7.1	33	17.7
More than 165,000 CHF/year	45	6.4	10	4.3	12	4.3	23	12.4
Education	811	100	274	100	313	100	224	100
Unfinished mandatory school	4	0.5	1	0.4	3	1.0	0	0
Mandatory school	72	8.9	13	4.7	47	15.0	12	5.4
Professional school	319	39.3	109	39.8	136	43.5	74	33.0
High school	53	6.5	21	7.7	21	6.7	11	4.9
University (BA/MA)	326	40.2	110	40.1	102	32.6	114	50.9
Ph.D.	37	4.6	20	7.3	4	1.3	13	5.8
Language	878	100	294	100	347	100	237	100
German	401	45.7	294	100	0	0	107	45.1
French	477	54.3	0	0	347	100.0	130	54.9
Total	878	100	294	33.5	347	39.5	237	27.0

N.B. ‘Couples’ include married and unmarried tenants; ‘alone’ includes divorced, widowed or separated tenants

**Table 8:** Respondent profiles for block 2: housing functions

Variable	N	Current (*t* − 1)		Current (*t* + 1)^a^		Ideal (*t* + 1)		ΔCurrent (*t, t* − 1)		ΔCurrent-Ideal (*t* + 1)		ΔFunctions (*t, t* − 1) ^b^		Gap (*t* + 1)^b^	
		Mean	SD	Mean	SD	Mean	SD	Mean	SD	Mean	SD	Mean	SD	Mean	SD
Property	878	2.65	1.234	2.71	1.211	3.63	1.164	0.06	1.056	− 0.92	1.402	0.58	0.886	1.02	1.284
Production, consumption	878	4.02	0.828	4.29	0.686	4.55	0.609	0.27	0.859	− 0.26	0.750	0.48	0.762	0.35	0.613
Impermanence	878	3.04	1.136	3.03	1.131	3.02	1.135	− 0.01	1.230	0.00	1.150	0.80	0.937	0.36	0.708
Status symbol	878	2.09	1.043	2.07	0.988	2.10	1.007	− 0.02	0.973	− 0.03	0.815	0.53	0.817	0.24	0.564
Security	878	3.48	1.041	3.61	0.947	3.47	0.988	0.14	1.034	0.14	0.850	0.62	0.840	0.18	0.482
Commodity	878	3.35	1.158	3.10	1.218	2.69	1.175	− 0.24	1.345	0.42	1.127	0.91	1.022	0.17	0.501
Self-representation	878	3.06	1.082	3.36	0.984	3.59	0.965	0.30	1.181	− 0.24	1.074	0.79	0.923	0.45	0.827
Shelter	878	3.58	1.045	3.95	0.909	4.17	0.923	0.37	1.029	− 0.22	0.800	0.67	0.865	0.34	0.627
Permanence	878	3.02	1.194	3.44	1.069	3.78	0.991	0.42	1.277	− 0.34	0.950	0.89	1.003	0.47	0.787

^a^Equivalent to current function at time *t*

^b^The variable ‘Gap’ measures the absolute difference at *t* + 1 between tenants’ current and ideal functions when the latter exceeds the former (Eq. 1); ‘ΔFunctions’ measures the absolute difference between tenants’ current functions at *t* and *t* − 1, whose average is used for running the ANOVA (Eq. 2; Table 6)

**Table 9:** Respondent profiles for block 3: trigger

Variable [0, 1]	Full sample		Opportunity		Problem-solving		Radical change	
	N	%	N	%	N	%	N	%
Total	875	100	323	100	216	100	336	100
Raise in salary	10	1.1	8	2.5	0	0	2	0.6
Retirement	12	1.4	5	1.5	2	0.9	5	1.5
Opportunity to rent^a^	107	12.2	85	26.3	8	3.7	14	4.2
Accessibility	14	1.6	7	2.2	5	2.3	2	0.6
New job location	49	5.6	9	2.8	9	4.2	31	9.2
Rental contract expiration	17	1.9	2	0.6	13	6.0	2	0.6
Interpersonal problems	13	1.5	3	0.9	8	3.7	2	0.6
Increasing lack of comfort	101	11.5	49	15.2	38	17.6	14	4.2
Divorce, separation, loss of partner	71	8.1	2	0.6	17	7.9	52	15.5
Move with partner	90	10.3	27	8.4	5	2.3	58	17.3
New child or household growth	97	11.1	30	9.3	14	6.5	53	15.8
Need for autonomy	27	3.1	7	2.2	4	1.9	16	4.8
Need for a radical change	22	2.5	5	1.5	2	0.9	15	4.5
Rent too high	56	6.4	13	4.0	29	13.4	14	4.2
Children leaving home	46	5.3	20	6.2	5	2.3	21	6.3
Leaving parent(s)’ home	10	1.1	1	0.3	0	0	9	2.7
Forced move^b^	66	7.5	24	7.4	27	12.5	15	4.5
Lack of space	50	5.7	23	7.1	22	10.2	5	1.5
Family (ageing, children)^c^	9	1.0	2	0.6	4	1.9	3	0.9
Change in life-location^d^	8	0.9	1	0.3	4	1.9	3	0.9

^a^Opportunity to rent another dwelling or acceptance from the cooperative

^b^Demolition, renovation

^c^Moves related to a change in household career (e.g. closer to the family when ageing, closer to schools for children)

^d^For example, moving to Switzerland

**Table 10:** Respondent profiles for block 4: residential satisfaction

Variable [1, 5]	Full sample			Strongly dissatisfied		Dissatisfied		Neither, nor		Satisfied		Strongly satisfied	
	N	Median	IQR	N	%	N	%	N	%	N	%	N	%
Satisfaction *t* − 1	878	4	1	55	6.3	62	7.1	52	5.9	347	39.5	362	41.2
Satisfaction *t*	878	4	1	78	8.9	130	14.8	150	17.1	328	37.4	192	21.9

**Table 12:** Overview of three binary logistic regressions of moving for a trigger type, depending on the event triggering the move

	Opportunity	Problem solving	Radical change
*Trigger event*			
Raise in salary	1.95** (0.831) [7.00]	− 18.69 (12,710.133) [0.00]	0.63 (1.092) [1.87]
Retirement	0.22 (0.639) [1.25]	0.91 (0.857) [2.48]	1.68* (0.954) [5.36]
Opportunity to rent	1.91*** (0.35) [6.76]	*ref*	0.12 (0.806) [1.13]
Accessibility	0.56 (0.593) [1.75]	1.93*** (0.668) [6.88]	0.22 (1.072) [1.25]
New job location	− 0.93** (0.449) [0.39]	1.02** (0.521) [2.78]	2.56*** (0.809) [12.92]
Rental contract expiration	− 1.46* (0.795) [0.23]	3.69*** (0.68) [40.22]	*ref*
Interpersonal problems	− 0.64 (0.706) [0.53]	2.99*** (0.678) [19.8]	0.31 (1.076) [1.36]
Increasing lack of comfort	0.50 (0.324) [1.65]	2.01*** (0.421) [7.46]	0.19 (0.806) [1.21]
Divorce, separation, loss of partner	− 2.98*** (0.762) [0.05]	1.36*** (0.461) [3.9]	3.02*** (0.799) [20.53]
Move with partner	− 0.29 (0.344) [0.75]	− 0.32 (0.589) [0.73]	2.61*** (0.784) [13.59]
New child or household growth	− 0.24 (0.337) [0.78]	0.74 (0.468) [2.09]	2.2*** (0.78) [9.03]
Need for autonomy	− 0.49 (0.508) [0.61]	0.77 (0.655) [2.15]	2.39*** (0.849) [10.91]
Need for radical change in life	− 0.66 (0.569) [0.51]	0.21 (0.828) [1.24]	2.78*** (0.881) [16.07]
Rent too high	− 0.64 (0.407) [0.53]	2.59*** (0.455) [13.29]	0.92 (0.814) [2.5]
Children leaving home	0.3 (0.392) [1.35]	0.41 (0.600) [1.51]	1.84** (0.809) [6.3]
Leaving parent's home	− 1.64 (1.085) [0.19]	− 18.69 (12,710.133) [0.00]	4.21*** (1.295) [67.5]
Forced move	*ref*	2.15*** (0.445) [8.57]	0.79 (0.808) [2.21]
Lack of space	0.40 (0.382) [1.49]	2.27*** (0.465) [9.72]	− 0.18 (0.888) [0.83]
Family (ageing, children)	− 0.69 (0.842) [0.50]	2.29*** (0.765) [9.90]	1.32 (1.033) [3.75]
Change in life-location	− 1.39 (1.099) [0.25]	2.52*** (0.797) [12.38]	1.5 (1.049) [4.5]
Constant	− 0.56** (0.256) [0.57]	− 2.52*** (0.368) [0.08]	− 2.01*** (0.753) [0.13]
N	878	878	878
− LL2	971	826	946
Improvement (Chi^2^)	Chi^2^ = 180.990, df = 19, *p* < 0.001***	Chi^2^ = 152.231, df = 19, *p* < 0.001***	Chi^2^ = 219.965, df = 19, *p* < 0.001***
Nagelkerke R^2^	0.255	0.237	0.302
Hosmer and Lemeshow test	*p* = 1	*p* = 1	*p* = 1
Classification accuracy	71.0%	76.9%	73.3%

Beta coefficients; ****p* < 0.01, ***p* < 0.05, **p* < 0.1; (standard error); [odds ratio]

**Table 13:** Bonferroni adjusted pairwise comparisons of mean change in function between current dwellings at *t* and *t* − 1 per trigger type

(I) trigger type	(J) trigger type	ΔMean (I–J)	SE	Sig.	95% C.I	
*Full sample*					L.B.	U.B.
Opportunity	Problem-solving	− 0.09	0.04	0.119	− 0.19	0.01
	Radical change	− 0.14	0.04	0.001***	− 0.23	− 0.04
Problem-solving	Opportunity	0.09	0.04	0.119	− 0.01	0.19
	Radical change	− 0.05	0.04	0.861	− 0.15	0.06
Radical change	Opportunity	0.14	0.04	0.001***	0.04	0.23
	Problem-solving	0.05	0.04	0.861	− 0.06	0.15
*Subsample ‘strongly satisfied’*						
Opportunity	Problem-solving	− 0.33	0.10	0.004***	− 0.57	− 0.09
	Radical change	− 0.36	0.09	0.000***	− 0.57	− 0.15
Problem-solving	Opportunity	0.33	0.10	0.004***	0.09	0.57
	Radical change	− 0.03	0.09	1.000	− 0.25	0.19
Radical change	Opportunity	0.36	0.09	0.000***	0.15	0.57
	Problem-solving	0.03	0.09	1.000	− 0.19	0.25

Based on observed means

The error term is Mean Square (Error) =  0.246 (full sample), Mean Square (Error) =  0.263 (subsample)

*L.B.* lower bound, *U.B.* upper bound

^***^*p* < 0.01, ***p* < 0.05, **p* < 0.1

**Table 14:** Ordinal logistic regression models of tenants’ agreement with the *ideal* housing functions according to changing socio-demographic characteristics and tenancy type

	Property	Impermanence	Status symbol	Shelter	Permanence
Threshold = 1	− 3.46*** (0.364) [0.03]	− 1.77*** (0.318) [0.17]	− 0.81*** (0.314) [0.45]	− 3.03*** (0.409) [0.05]	− 3.58*** (0.417) [0.03]
Threshold = 2	− 1.73*** (0.319) [0.18]	− 0.26 (0.306) [0.77]	0.78** (0.314) [2.18]	− 1.81*** (0.346) [0.16]	− 1.61*** (0.323) [0.20]
Threshold = 3	− 0.60* (0.311) [0.55]	1.03*** (0.309) [2.80]	2.27*** (0.332) [9.67]	− 0.83** (0.328) [0.43]	− 0.11 (0.312) [0.90]
Threshold = 4	0.99*** (0.313) [2.69]	2.80*** (0.330) [16.42]	4.27*** (0.453) [71.70]	1.38*** (0.331) [3.98]	1.74*** (0.321) [5.70]
*Household type (ref. cat. Middle-aged couple with children living at home)*					
Young single	1.75*** (0.387) [5.73]	0.34 (0.354) [1.41]	0.73** (0.358) [2.08]	0.54 (0.378) [1.71]	0.03 (0.360) [1.03]
Young couples without children	0.91*** (0.31) [2.49]	0.15 (0.300) [1.17]	0.45 (0.304) [1.57]	1.36*** (0.338) [3.90]	0.78** (0.309) [2.18]
Young couples with children	0.87** (0.411) [2.39]	0.27 (0.398) [1.31]	0.36 (0.403) [1.44]	1.34*** (0.468) [3.81]	0.40 (0.410) [1.50]
Middle-aged single	0.85*** (0.296) [2.34]	− 0.07 (0.288) [0.94]	− 0.37 (0.297) [0.69]	0.67** (0.310) [1.96]	0.10 (0.294) [1.11]
Middle-aged couples without children	0.46 (0.330) [1.59]	0.46 (0.324) [1.59]	− 0.34 (0.333) [0.71]	0.07 (0.343) [1.07]	− 0.14 (0.330) [0.87]
Middle-aged alone without children	0.03 (0.344) [1.03]	0.88*** (0.345) [2.42]	0.29 (0.349) [1.33]	0.05 (0.363) [1.06]	− 0.31 (0.349) [0.73]
Middle-aged couple with children not living at home	− 0.3 (0.376) [0.74]	0.44 (0.375) [1.55]	− 0.6 (0.395) [0.55]	− 0.93** (0.393) [0.39]	− 1.00*** (0.382) [0.37]
Middle-aged alone with children living at home	0.17 (0.339) [1.18]	0.47 (0.336) [1.59]	− 0.56 (0.351) [0.57]	0.18 (0.357) [1.19]	− 0.29 (0.342) [0.74]
Middle-aged alone with children not living at home	0.63 (0.409) [1.88]	0.69* (0.403) [1.98]	− 0.58 (0.418) [0.56]	0.91** (0.444) [2.48]	0.49 (0.417) [1.63]
Other middle-aged couples	0.33 (0.600) [1.39]	0.25 (0.593) [1.29]	0.56 (0.602) [1.75]	0.46 (0.634) [1.58]	1.11* (0.630) [3.03]
Older couple	− 0.78* (0.418) [0.46]	0.25 (0.417) [1.29]	− 0.2 (0.429) [0.82]	− 0.22 (0.439) [0.80]	− 0.06 (0.425) [0.94]
Older alone	− 0.21 (0.431) [0.81]	0.60 (0.431) [1.82]	− 0.03 (0.442) [0.97]	0.46 (0.457) [1.59]	0.04 (0.440) [1.04]
*Employment (ref. cat. Full-time 80–100%)*					
Part-time < 80%	− 0.23 (0.214) [0.79]	0.05 (0.211) [1.05]	− 0.7*** (0.220) [0.50]	0.37 (0.229) [1.45]	0.30 (0.217) [1.35]
Housewife/househusband	− 0.08 (0.552) [0.92]	0.16 (0.547) [1.17]	0.05 (0.557) [1.05]	1.23 (0.631) [3.43]	1.20** (0.574) [3.31]
Student or apprenticeship	− 0.98 (0.719) [0.38]	− 0.16 (0.71) [0.85]	0.07 (0.715) [1.07]	0.15 (0.814) [1.16]	− 0.58 (0.725) [0.56]
Unemployed	− 1.28** (0.518) [0.28]	0.97* (0.519) [2.64]	0.31 (0.524) [1.36]	0.14 (0.550) [1.15]	− 0.44 (0.523) [0.65]
Retired	− 0.02 (0.374) [0.98]	0.43 (0.373) [1.54]	− 0.22 (0.384) [0.80]	− 0.11 (0.393) [0.9]	0.44 (0.381) [1.55]
*Salary (ref. cat. Less than 60,000 CHF/year)*					
60,000–88,000 CHF/year	− 0.51** (0.208) [0.60]	− 0.23 (0.204) [0.79]	− 0.24 (0.211) [0.79]	0.21 (0.22) [1.23]	0.01 (0.21) [1.01]
88,001–120,000 CHF/year	− 0.22 (0.242) [0.8]	− 0.2 (0.238) [0.82]	− 0.03 (0.243) [0.97]	0.1 (0.256) [1.11]	− 0.16 (0.244) [0.85]
120,001–164,999 CHF/year	− 0.51 (0.312) [0.6]	0.2 (0.307) [1.23]	− 0.08 (0.313) [0.92]	− 0.18 (0.326) [0.84]	− 0.26 (0.313) [0.77]
> 165,000 CHF/year	0.28 (0.383) [1.33]	0.28 (0.37) [1.33]	0.36 (0.375) [1.43]	0.13 (0.396) [1.14]	− 0.42 (0.377) [0.66]
*Education (ref. cat. University (BA/MA)*					
Unfinished school	− 1.49 (0.937) [0.23]	− 0.42 (0.934) [0.66]	0.04 (0.95) [1.04]	− 1.6* (0.949) [0.2]	0.21 (0.961) [1.24]
Mandatory school	− 0.37 (0.304) [0.69]	0.06 (0.302) [1.06]	0.14 (0.309) [1.14]	0.61* (0.328) [1.84]	0.07 (0.308) [1.07]
Professional school	− 0.14 (0.174) [0.87]	0.13 (0.171) [1.14]	− 0.14 (0.175) [0.87]	0.26 (0.184) [1.3]	− 0.01 (0.175) [0.99]
High school	− 0.72** (0.312) [0.49]	− 0.24 (0.308) [0.79]	− 0.33 (0.317) [0.72]	0.14 (0.328) [1.15]	0.38 (0.317) [1.46]
PhD	0.09 (0.357) [1.09]	− 0.83** (0.352) [0.44]	− 0.39 (0.360) [0.68]	− 0.31 (0.372) [0.74]	0.18 (0.359) [1.19]
*Owner (ref. cat. SCHL)*					
ABZ	0.11 (0.175) [1.12]	− 0.21 (0.173) [0.81]	0.46*** (0.179) [1.58]	0.9*** (0.189) [2.47]	1.06*** (0.182) [2.89]
Mobiliar	0.41** (0.194) [1.51]	0.41** (0.191) [1.51]	0.34* (0.194) [1.40]	0.37* (0.203) [1.44]	0.24 (0.193) [1.28]
N (valid)	651	651	651	651	651
Initial − 2 log likelihood	1512	1550	1346	1189	1384
Model − 2 log likelihood	1402	1495	1294	1106	1306
Improvement (Chi^2^)	Chi^2^ = 109.951, df = 28, *p* < 0.001***	Chi^2^ = 55.278, df = 28, *p* < 0.01***	Chi^2^ = 52.127, df = 28, *p* < 0.01***	Chi^2^ = 83.123, df = 28, *p* < 0.001***	Chi^2^ = 77.172, df = 28, *p* < 0.001***
Nagelkerke R^2^	0.164	0.085	0.083	0.133	0.120
Test of parallel lines	Chi^2^ = 73.910, df = 84, *p* = 0.776	Chi^2^ = 82.645, df = 84, *p* = 0.521	Chi^2^ = 94.739, df = 84, *p* = 0.199	Chi^2^ = 101.058, df = 84, *p* = 0.099	Chi^2^ = 105.963, df = 84, *p* = 0.052

Beta coefficients; ****p* < 0.01, ***p* < 0.05, **p* < 0.1; (standard error); [odds ratio]

## Footnotes

1 IBM SPSS Statistics 26. https://www.ibm.com/analytics/spss-statistics-software.

2 This choice was supported by the assumption that the distance between categories of satisfaction (e.g. ‘strongly dissatisfied’ and ‘dissatisfied’) is not always equal.

3 In order to perform the transformations illustrated in point 1 and point 3, the distance between adjacent answer categories (i.e. ratings on each housing function) was assumed to be equal.

4 We can attribute this to two elements: first, cooperative systems often request tenants’ participation in surveys; second, the invitation letter to the cooperatives included the signature of the cooperatives’ directors, which was not present in those from Swiss Mobiliar. In fact, the presence of its logo could have biased the responses, as (1) Mobiliar is an insurance company and (2) the tenants rent through technical administrations and are often unaware of their dwelling owner’s identity.

5 The percentages are ‘valid percentages’, which exclude the missing values (or those tenants who did not answer the question) from the total.

6 To check the criterion of parsimony in the model, we computed the Akaike information criterion (AIC). Results show that the AIC is lower in the partial models (satisfaction: 467; function: 4750) than in the full one (4797), meaning that the full model performs less well than the partial one. However, the goal of our analysis in this case was *exploratory* rather than predictive.
